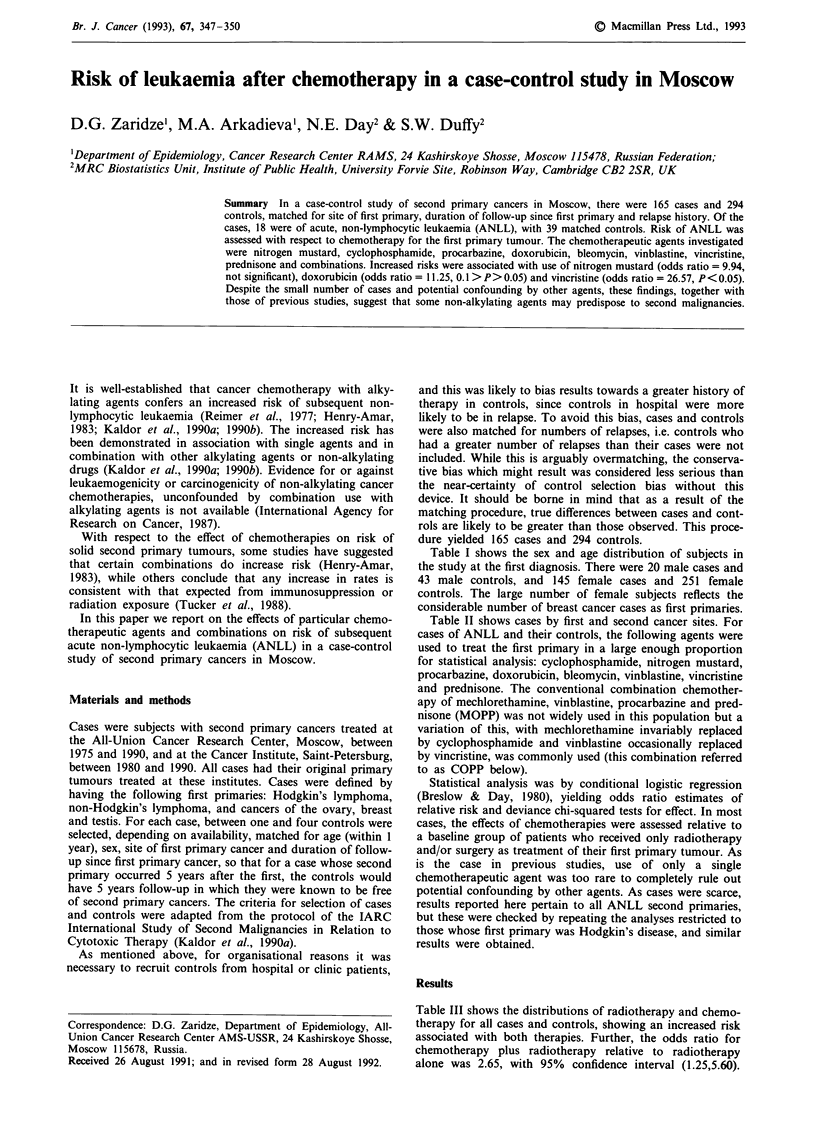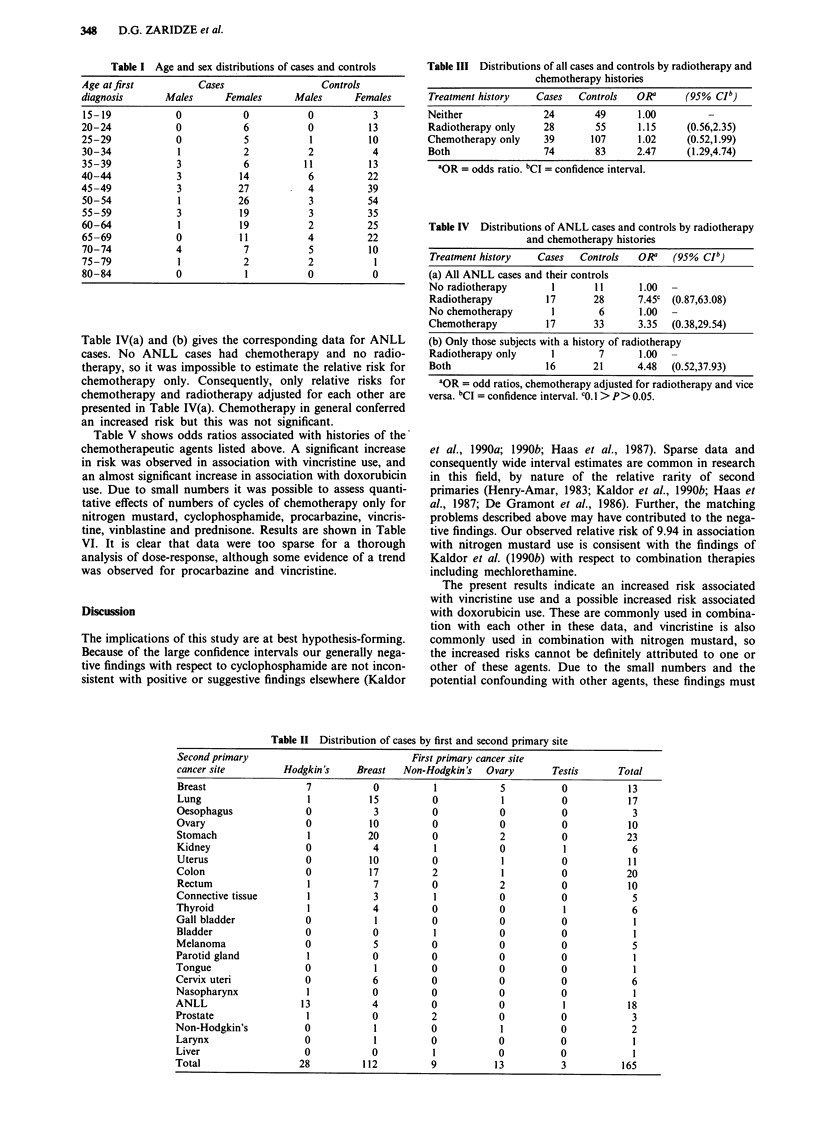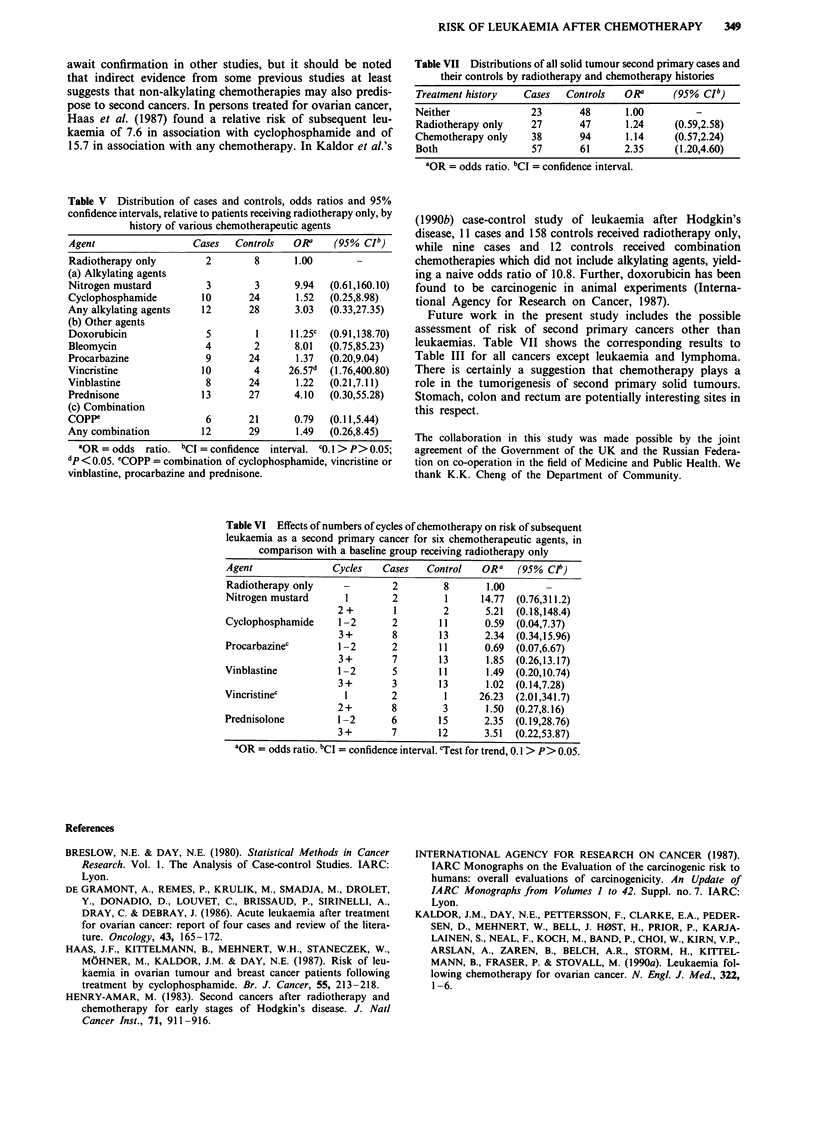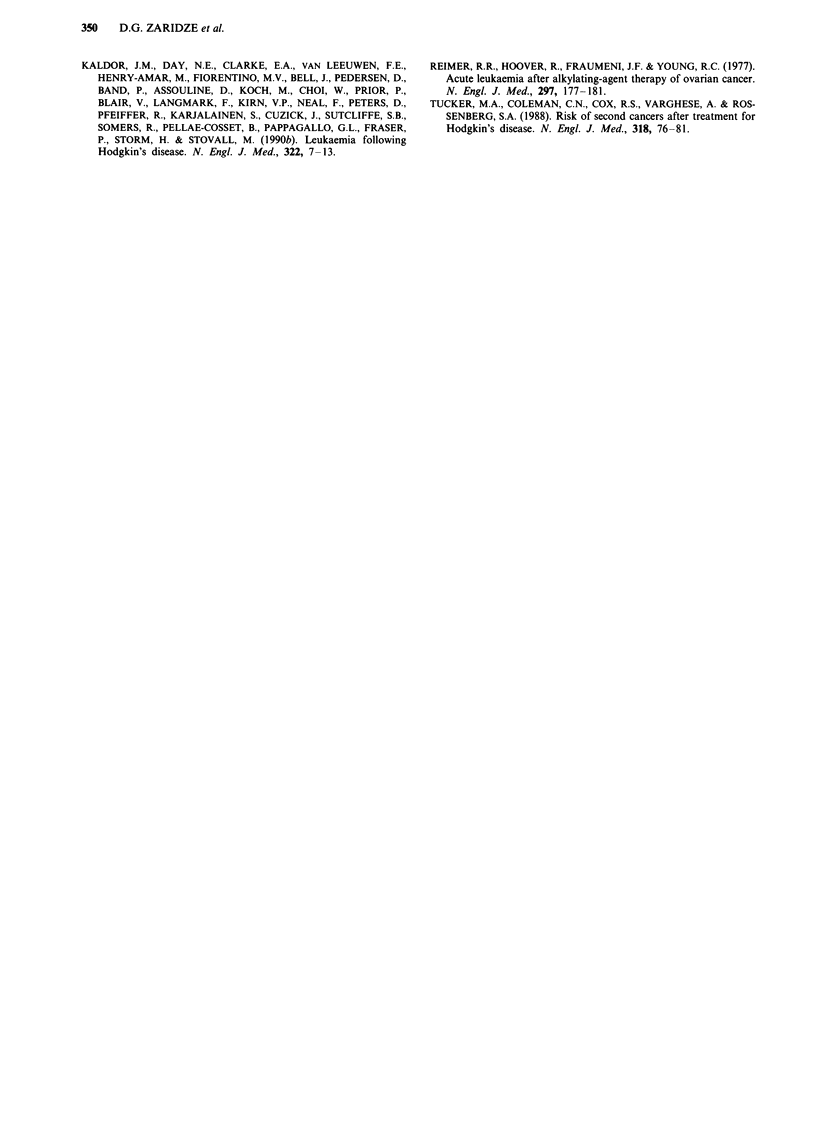# Risk of leukaemia after chemotherapy in a case-control study in Moscow.

**DOI:** 10.1038/bjc.1993.63

**Published:** 1993-02

**Authors:** D. G. Zaridze, M. A. Arkadieva, N. E. Day, S. W. Duffy

**Affiliations:** Department of Epidemiology, Cancer Research Center RAMS, Moscow, Russian Federation.

## Abstract

In a case-control study of second primary cancers in Moscow, there were 165 cases and 294 controls, matched for site of first primary, duration of follow-up since first primary and relapse history. Of the cases, 18 were of acute, non-lymphocytic leukaemia (ANLL), with 39 matched controls. Risk of ANLL was assessed with respect to chemotherapy for the first primary tumour. The chemotherapeutic agents investigated were nitrogen mustard, cyclophosphamide, procarbazine, doxorubicin, bleomycin, vinblastine, vincristine, prednisone and combinations. Increased risks were associated with use of nitrogen mustard (odds ratio = 9.94, not significant), doxorubicin (odds ratio = 11.25, 0.1 > P > 0.05) and vincristine (odds ratio = 26.57, P < 0.05). Despite the small number of cases and potential confounding by other agents, these findings, together with those of previous studies, suggest that some non-alkylating agents may predispose to second malignancies.


*Br. J. Cancer* (1993), **67**, 347–350 © Macmillan Press Ltd., 1993

### Risk of leukaemia after chemotherapy in a case-control study in Moscow

D.G. Zaridze[1], M.A. Arkadieva[1], N.E. Day[2] & S.W. Duffy[2]

[1]*Department of Epidemiology, Cancer Research Center RAMS, 24 Kashirskoye Shosse, Moscow 115478, Russian Federation;* [2]*MRC Biostatistics Unit, Institute of Public Health, University Forvie Site, Robinson Way, Cambridge CB2 2SR, UK*

**Summary** In a case-control study of second primary cancers in Moscow, there were 165 cases and 294 controls, matched for site of first primary, duration of follow-up since first primary and relapse history. Of the cases, 18 were of acute, non-lymphocytic leukaemia (ANLL), with 39 matched controls. Risk of ANLL was assessed with respect to chemotherapy for the first primary tumour. The chemotherapeutic agents investigated were nitrogen mustard, cyclophosphamide, procarbazine, doxorubicin, bleomycin, vinblastine, vincristine, prednisone and combinations. Increased risks were associated with use of nitrogen mustard (odds ratio = 9.94, not significant), doxorubicin (odds ratio = 11.25, $0.1 > P > 0.05$) and vincristine (odds ratio = 26.57, $P < 0.05$). Despite the small number of cases and potential confounding by other agents, these findings, together with those of previous studies, suggest that some non-alkylating agents may predispose to second malignancies.

It is well-established that cancer chemotherapy with alkylating agents confers an increased risk of subsequent non-lymphocytic leukaemia (Reimer *et al.*, 1977; Henry-Amar, 1983; Kaldor *et al.*, 1990*a*; 1990*b*). The increased risk has been demonstrated in association with single agents and in combination with other alkylating agents or non-alkylating drugs (Kaldor *et al.*, 1990*a*; 1990*b*). Evidence for or against leukaemogenicity or carcinogenicity of non-alkylating cancer chemotherapies, unconfounded by combination use with alkylating agents is not available (International Agency for Research on Cancer, 1987).

With respect to the effect of chemotherapies on risk of solid second primary tumours, some studies have suggested that certain combinations do increase risk (Henry-Amar, 1983), while others conclude that any increase in rates is consistent with that expected from immunosuppression or radiation exposure (Tucker *et al.*, 1988).

In this paper we report on the effects of particular chemotherapeutic agents and combinations on risk of subsequent acute non-lymphocytic leukaemia (ANLL) in a case-control study of second primary cancers in Moscow.

### Materials and methods

Cases were subjects with second primary cancers treated at the All-Union Cancer Research Center, Moscow, between 1975 and 1990, and at the Cancer Institute, Saint-Petersburg, between 1980 and 1990. All cases had their original primary tumours treated at these institutes. Cases were defined by having the following first primaries: Hodgkin's lymphoma, non-Hodgkin's lymphoma, and cancers of the ovary, breast and testis. For each case, between one and four controls were selected, depending on availability, matched for age (within 1 year), sex, site of first primary cancer and duration of follow-up since first primary cancer, so that for a case whose second primary occurred 5 years after the first, the controls would have 5 years follow-up in which they were known to be free of second primary cancers. The criteria for selection of cases and controls were adapted from the protocol of the IARC International Study of Second Malignancies in Relation to Cytotoxic Therapy (Kaldor *et al.*, 1990*a*).

As mentioned above, for organisational reasons it was necessary to recruit controls from hospital or clinic patients, and this was likely to bias results towards a greater history of therapy in controls, since controls in hospital were more likely to be in relapse. To avoid this bias, cases and controls were also matched for numbers of relapses, i.e. controls who had a greater number of relapses than their cases were not included. While this is arguably overmatching, the conservative bias which might result was considered less serious than the near-certainty of control selection bias without this device. It should be borne in mind that as a result of the matching procedure, true differences between cases and controls are likely to be greater than those observed. This procedure yielded 165 cases and 294 controls.

Table I shows the sex and age distribution of subjects in the study at the first diagnosis. There were 20 male cases and 43 male controls, and 145 female cases and 251 female controls. The large number of female subjects reflects the considerable number of breast cancer cases as first primaries.

Table II shows cases by first and second cancer sites. For cases of ANLL and their controls, the following agents were used to treat the first primary in a large enough proportion for statistical analysis: cyclophosphamide, nitrogen mustard, procarbazine, doxorubicin, bleomycin, vinblastine, vincristine and prednisone. The conventional combination chemotherapy of mechlorethamine, vinblastine, procarbazine and prednisone (MOPP) was not widely used in this population but a variation of this, with mechlorethamine invariably replaced by cyclophosphamide and vinblastine occasionally replaced by vincristine, was commonly used (this combination referred to as COPP below).

Statistical analysis was by conditional logistic regression (Breslow & Day, 1980), yielding odds ratio estimates of relative risk and deviance chi-squared tests for effect. In most cases, the effects of chemotherapies were assessed relative to a baseline group of patients who received only radiotherapy and/or surgery as treatment of their first primary tumour. As is the case in previous studies, use of only a single chemotherapeutic agent was too rare to completely rule out potential confounding by other agents. As cases were scarce, results reported here pertain to all ANLL second primaries, but these were checked by repeating the analyses restricted to those whose first primary was Hodgkin's disease, and similar results were obtained.

### Results

Table III shows the distributions of radiotherapy and chemotherapy for all cases and controls, showing an increased risk associated with both therapies. Further, the odds ratio for chemotherapy plus radiotherapy relative to radiotherapy alone was 2.65, with 95% confidence interval (1.25,5.60).

Correspondence: D.G. Zaridze, Department of Epidemiology, All-Union Cancer Research Center AMS-USSR, 24 Kashirskoye Shosse, Moscow 115678, Russia.
Received 26 August 1991; and in revised form 28 August 1992.

**Table I** Age and sex distributions of cases and controls

| Age at first diagnosis | Cases Males | Cases Females | Controls Males | Controls Females |
|---|---|---|---|---|
| 15-19 | 0 | 0 | 0 | 3 |
| 20-24 | 0 | 6 | 0 | 13 |
| 25-29 | 0 | 5 | 1 | 10 |
| 30-34 | 1 | 2 | 2 | 4 |
| 35-39 | 3 | 6 | 11 | 13 |
| 40-44 | 3 | 14 | 6 | 22 |
| 45-49 | 3 | 27 | 4 | 39 |
| 50-54 | 1 | 26 | 3 | 54 |
| 55-59 | 3 | 19 | 3 | 35 |
| 60-64 | 1 | 19 | 2 | 25 |
| 65-69 | 0 | 11 | 4 | 22 |
| 70-74 | 4 | 7 | 5 | 10 |
| 75-79 | 1 | 2 | 2 | 1 |
| 80-84 | 0 | 1 | 0 | 0 |

**Table III** Distributions of all cases and controls by radiotherapy and chemotherapy histories

| Treatment history | Cases | Controls | OR[a] | (95% CI[b]) |
|---|---|---|---|---|
| Neither | 24 | 49 | 1.00 | – |
| Radiotherapy only | 28 | 55 | 1.15 | (0.56,2.35) |
| Chemotherapy only | 39 | 107 | 1.02 | (0.52,1.99) |
| Both | 74 | 83 | 2.47 | (1.29,4.74) |

[a]OR = odds ratio. [b]CI = confidence interval.

**Table IV** Distributions of ANLL cases and controls by radiotherapy and chemotherapy histories

| Treatment history | Cases | Controls | OR[a] | (95% CI[b]) |
|---|---|---|---|---|
| (a) All ANLL cases and their controls | | | | |
| No radiotherapy | 1 | 11 | 1.00 | – |
| Radiotherapy | 17 | 28 | 7.45[c] | (0.87,63.08) |
| No chemotherapy | 1 | 6 | 1.00 | – |
| Chemotherapy | 17 | 33 | 3.35 | (0.38,29.54) |
| (b) Only those subjects with a history of radiotherapy | | | | |
| Radiotherapy only | 1 | 7 | 1.00 | – |
| Both | 16 | 21 | 4.48 | (0.52,37.93) |

[a]OR = odd ratios, chemotherapy adjusted for radiotherapy and vice versa. [b]CI = confidence interval. [c]$0.1 > P > 0.05$.

Table IV(a) and (b) gives the corresponding data for ANLL cases. No ANLL cases had chemotherapy and no radiotherapy, so it was impossible to estimate the relative risk for chemotherapy only. Consequently, only relative risks for chemotherapy and radiotherapy adjusted for each other are presented in Table IV(a). Chemotherapy in general conferred an increased risk but this was not significant.

Table V shows odds ratios associated with histories of the chemotherapeutic agents listed above. A significant increase in risk was observed in association with vincristine use, and an almost significant increase in association with doxorubicin use. Due to small numbers it was possible to assess quantitative effects of numbers of cycles of chemotherapy only for nitrogen mustard, cyclophosphamide, procarbazine, vincristine, vinblastine and prednisone. Results are shown in Table VI. It is clear that data were too sparse for a thorough analysis of dose-response, although some evidence of a trend was observed for procarbazine and vincristine.

### Discussion

The implications of this study are at best hypothesis-forming. Because of the large confidence intervals our generally negative findings with respect to cyclophosphamide are not inconsistent with positive or suggestive findings elsewhere (Kaldor et al., 1990a; 1990b; Haas et al., 1987). Sparse data and consequently wide interval estimates are common in research in this field, by nature of the relative rarity of second primaries (Henry-Amar, 1983; Kaldor et al., 1990b; Haas et al., 1987; De Gramont et al., 1986). Further, the matching problems described above may have contributed to the negative findings. Our observed relative risk of 9.94 in association with nitrogen mustard use is consisent with the findings of Kaldor et al. (1990b) with respect to combination therapies including mechlorethamine.

The present results indicate an increased risk associated with vincristine use and a possible increased risk associated with doxorubicin use. These are commonly used in combination with each other in these data, and vincristine is also commonly used in combination with nitrogen mustard, so the increased risks cannot be definitely attributed to one or other of these agents. Due to the small numbers and the potential confounding with other agents, these findings must

**Table II** Distribution of cases by first and second primary site

| Second primary cancer site | First primary cancer site: Hodgkin's | Breast | Non-Hodgkin's | Ovary | Testis | Total |
|---|---|---|---|---|---|---|
| Breast | 7 | 0 | 1 | 5 | 0 | 13 |
| Lung | 1 | 15 | 0 | 1 | 0 | 17 |
| Oesophagus | 0 | 3 | 0 | 0 | 0 | 3 |
| Ovary | 0 | 10 | 0 | 0 | 0 | 10 |
| Stomach | 1 | 20 | 0 | 2 | 0 | 23 |
| Kidney | 0 | 4 | 1 | 0 | 1 | 6 |
| Uterus | 0 | 10 | 0 | 1 | 0 | 11 |
| Colon | 0 | 17 | 2 | 1 | 0 | 20 |
| Rectum | 1 | 7 | 0 | 2 | 0 | 10 |
| Connective tissue | 1 | 3 | 1 | 0 | 0 | 5 |
| Thyroid | 1 | 4 | 0 | 0 | 1 | 6 |
| Gall bladder | 0 | 1 | 0 | 0 | 0 | 1 |
| Bladder | 0 | 0 | 1 | 0 | 0 | 1 |
| Melanoma | 0 | 5 | 0 | 0 | 0 | 5 |
| Parotid gland | 1 | 0 | 0 | 0 | 0 | 1 |
| Tongue | 0 | 1 | 0 | 0 | 0 | 1 |
| Cervix uteri | 0 | 6 | 0 | 0 | 0 | 6 |
| Nasopharynx | 1 | 0 | 0 | 0 | 0 | 1 |
| ANLL | 13 | 4 | 0 | 0 | 1 | 18 |
| Prostate | 1 | 0 | 2 | 0 | 0 | 3 |
| Non-Hodgkin's | 0 | 1 | 0 | 1 | 0 | 2 |
| Larynx | 0 | 1 | 0 | 0 | 0 | 1 |
| Liver | 0 | 0 | 1 | 0 | 0 | 1 |
| Total | 28 | 112 | 9 | 13 | 3 | 165 |

await confirmation in other studies, but it should be noted that indirect evidence from some previous studies at least suggests that non-alkylating chemotherapies may also predispose to second cancers. In persons treated for ovarian cancer, Haas *et al.* (1987) found a relative risk of subsequent leukaemia of 7.6 in association with cyclophosphamide and of 15.7 in association with any chemotherapy. In Kaldor *et al.*'s

**Table V** Distribution of cases and controls, odds ratios and 95% confidence intervals, relative to patients receiving radiotherapy only, by history of various chemotherapeutic agents

| *Agent* | *Cases* | *Controls* | *OR*[a] | *(95% CI*[b]*)* |
|---|---|---|---|---|
| Radiotherapy only | 2 | 8 | 1.00 | – |
| (a) Alkylating agents | | | | |
| Nitrogen mustard | 3 | 3 | 9.94 | (0.61,160.10) |
| Cyclophosphamide | 10 | 24 | 1.52 | (0.25,8.98) |
| Any alkylating agents | 12 | 28 | 3.03 | (0.33,27.35) |
| (b) Other agents | | | | |
| Doxorubicin | 5 | 1 | 11.25[c] | (0.91,138.70) |
| Bleomycin | 4 | 2 | 8.01 | (0.75,85.23) |
| Procarbazine | 9 | 24 | 1.37 | (0.20,9.04) |
| Vincristine | 10 | 4 | 26.57[d] | (1.76,400.80) |
| Vinblastine | 8 | 24 | 1.22 | (0.21,7.11) |
| Prednisone | 13 | 27 | 4.10 | (0.30,55.28) |
| (c) Combination | | | | |
| COPP[e] | 6 | 21 | 0.79 | (0.11,5.44) |
| Any combination | 12 | 29 | 1.49 | (0.26,8.45) |

[a]OR = odds ratio. [b]CI = confidence interval. [c]$0.1 > P > 0.05$; [d]$P < 0.05$. [e]COPP = combination of cyclophosphamide, vincristine or vinblastine, procarbazine and prednisone.

**Table VII** Distributions of all solid tumour second primary cases and their controls by radiotherapy and chemotherapy histories

| *Treatment history* | *Cases* | *Controls* | *OR*[a] | *(95% CI*[b]*)* |
|---|---|---|---|---|
| Neither | 23 | 48 | 1.00 | – |
| Radiotherapy only | 27 | 47 | 1.24 | (0.59,2.58) |
| Chemotherapy only | 38 | 94 | 1.14 | (0.57,2.24) |
| Both | 57 | 61 | 2.35 | (1.20,4.60) |

[a]OR = odds ratio. [b]CI = confidence interval.

(1990*b*) case-control study of leukaemia after Hodgkin's disease, 11 cases and 158 controls received radiotherapy only, while nine cases and 12 controls received combination chemotherapies which did not include alkylating agents, yielding a naive odds ratio of 10.8. Further, doxorubicin has been found to be carcinogenic in animal experiments (International Agency for Research on Cancer, 1987).

Future work in the present study includes the possible assessment of risk of second primary cancers other than leukaemias. Table VII shows the corresponding results to Table III for all cancers except leukaemia and lymphoma. There is certainly a suggestion that chemotherapy plays a role in the tumorigenesis of second primary solid tumours. Stomach, colon and rectum are potentially interesting sites in this respect.

The collaboration in this study was made possible by the joint agreement of the Government of the UK and the Russian Federation on co-operation in the field of Medicine and Public Health. We thank K.K. Cheng of the Department of Community.

**Table VI** Effects of numbers of cycles of chemotherapy on risk of subsequent leukaemia as a second primary cancer for six chemotherapeutic agents, in comparison with a baseline group receiving radiotherapy only

| *Agent* | *Cycles* | *Cases* | *Control* | *OR*[a] | *(95% CI*[b]*)* |
|---|---|---|---|---|---|
| Radiotherapy only | – | 2 | 8 | 1.00 | – |
| Nitrogen mustard | 1 | 2 | 1 | 14.77 | (0.76,311.2) |
| | 2+ | 1 | 2 | 5.21 | (0.18,148.4) |
| Cyclophosphamide | 1-2 | 2 | 11 | 0.59 | (0.04,7.37) |
| | 3+ | 8 | 13 | 2.34 | (0.34,15.96) |
| Procarbazine[c] | 1-2 | 2 | 11 | 0.69 | (0.07,6.67) |
| | 3+ | 7 | 13 | 1.85 | (0.26,13.17) |
| Vinblastine | 1-2 | 5 | 11 | 1.49 | (0.20,10.74) |
| | 3+ | 3 | 13 | 1.02 | (0.14,7.28) |
| Vincristine[c] | 1 | 2 | 1 | 26.23 | (2.01,341.7) |
| | 2+ | 8 | 3 | 1.50 | (0.27,8.16) |
| Prednisolone | 1-2 | 6 | 15 | 2.35 | (0.19,28.76) |
| | 3+ | 7 | 12 | 3.51 | (0.22,53.87) |

[a]OR = odds ratio. [b]CI = confidence interval. [c]Test for trend, $0.1 > P > 0.05$.

### References

BRESLOW, N.E. & DAY, N.E. (1980). *Statistical Methods in Cancer Research*. Vol. 1. The Analysis of Case-control Studies. IARC: Lyon.

DE GRAMONT, A., REMES, P., KRULIK, M., SMADJA, M., DROLET, Y., DONADIO, D., LOUVET, C., BRISSAUD, P., SIRINELLI, A., DRAY, C. & DEBRAY, J. (1986). Acute leukaemia after treatment for ovarian cancer: report of four cases and review of the literature. *Oncology*, **43**, 165-172.

HAAS, J.F., KITTELMANN, B., MEHNERT, W.H., STANECZEK, W., MÖHNER, M., KALDOR, J.M. & DAY, N.E. (1987). Risk of leukaemia in ovarian tumour and breast cancer patients following treatment by cyclophosphamide. *Br. J. Cancer*, **55**, 213-218.

HENRY-AMAR, M. (1983). Second cancers after radiotherapy and chemotherapy for early stages of Hodgkin's disease. *J. Natl Cancer Inst.*, **71**, 911-916.

INTERNATIONAL AGENCY FOR RESEARCH ON CANCER (1987). IARC Monographs on the Evaluation of the carcinogenic risk to humans: overall evaluations of carcinogenicity. *An Update of IARC Monographs from Volumes 1 to 42*. Suppl. no. 7. IARC: Lyon.

KALDOR, J.M., DAY, N.E., PETTERSSON, F., CLARKE, E.A., PEDERSEN, D., MEHNERT, W., BELL, J. HØST, H., PRIOR, P., KARJALAINEN, S., NEAL, F., KOCH, M., BAND, P., CHOI, W., KIRN, V.P., ARSLAN, A., ZAREN, B., BELCH, A.R., STORM, H., KITTELMANN, B., FRASER, P. & STOVALL, M. (1990*a*). Leukaemia following chemotherapy for ovarian cancer. *N. Engl. J. Med.*, **322**, 1-6.

KALDOR, J.M., DAY, N.E., CLARKE, E.A., VAN LEEUWEN, F.E., HENRY-AMAR, M., FIORENTINO, M.V., BELL, J., PEDERSEN, D., BAND, P., ASSOULINE, D., KOCH, M., CHOI, W., PRIOR, P., BLAIR, V., LANGMARK, F., KIRN, V.P., NEAL, F., PETERS, D., PFEIFFER, R., KARJALAINEN, S., CUZICK, J., SUTCLIFFE, S.B., SOMERS, R., PELLAE-COSSET, B., PAPPAGALLO, G.L., FRASER, P., STORM, H. & STOVALL, M. (1990*b*). Leukaemia following Hodgkin's disease. *N. Engl. J. Med.*, **322,** 7–13.

REIMER, R.R., HOOVER, R., FRAUMENI, J.F. & YOUNG, R.C. (1977). Acute leukaemia after alkylating-agent therapy of ovarian cancer. *N. Engl. J. Med.*, **297,** 177–181.

TUCKER, M.A., COLEMAN, C.N., COX, R.S., VARGHESE, A. & ROSSENBERG, S.A. (1988). Risk of second cancers after treatment for Hodgkin's disease. *N. Engl. J. Med.*, **318,** 76–81.